# How May Longer Console Times Influence Outcomes after Robot-Assisted Radical Prostatectomy (RARP)?

**DOI:** 10.3390/jcm12124022

**Published:** 2023-06-13

**Authors:** Mahmoud Farzat, Mohamed Elsherif, Florian M. Wagenlehner

**Affiliations:** 1 Department of Urology and Robotic Urology, Diakonie Klinikum Siegen, Academic Teaching Hospital, University of Bonn, 53127 Bonn, Germany; 2 Department of Urology, Pediatric Urology and Andrology, Justus-Liebig University of Giessen, 35390 Giessen, Germany; florian.wagenlehner@chiru.med.uni-giessen.de; 3 Department of Epidemiology and Public Health, Faculty of Health Sciences, American University of Beirut, Beirut 2020, Lebanon

**Keywords:** prostate cancer, robot-assisted radical prostatectomy (RARP), console time

## Abstract

Longer operating time in radical prostatectomy may increase the risk of perioperative complications. Various factors such as cancer extent, the procedure’s level of difficulty, habitus and previous surgeries may lengthen robot-assisted radical prostatectomy (RARP) and therefore compromise outcomes. Objective: this study investigates the influence of operating time on outcomes after RARP in real life settings in a monocentric single surgeon study. Methods: a total of 500 sequential patients who were operated on between April 2019 and August 2022 were involved. Men were allocated to three groups short (*n* = 157; 31.4%), under or equal to 120 min; average (*n* = 255; 51%), between 121 and 180 min; long (*n* = 88; 17.6%), above 180 min console time. Demographic, baseline and perioperative data were analyzed and compared between groups. Univariate logistic regression was completed to investigate the association between console time and outcomes and to predict factors which may prolong surgery. Results: hospital stay and catheter days were significantly longer in group 3 with medians of 6 and 7 days (*p* < 0.001 and <0.001, respectively). Those findings were confirmed in univariate analysis, with *p* = 0.012 for catheter days and *p* < 0.001 for hospital stay. Moreover, major complications were higher in patients with longer procedures, at *p* = 0.008. Prostate volume was the only predictor of a prolonged console time (*p* = 0.005). Conclusion: RARP is a safe procedure and most patients will be discharged uneventfully. Yet, a longer console time is associated with a longer hospital stay, longer catheter days and major complications. Caution has to be taken in the large prostate to avoid longer procedures, which may prevent postoperative adverse events.

## 1. Introduction

Longer operating time in radical prostatectomy is known to increase the risk of perioperative complications, especially venous thromboembolism (VTE) [1,2]. It is also reported to be associated with the development of symptomatic lymphoceles [3]. In the early days of robotic introduction in prostate surgery, operating time was longer compared to that of open retropubic radical prostatectomy [4,5]. As part of the learning curve, urologists need longer times to complete the procedures [5,6]. Cancer extent and the procedure’s level of difficulty as well as prostate volume, habitus and previous abdominal surgery play a contributing role in prolonging robot-assisted radical prostatectomy (RARP) [7]. This imposes the need to investigate the impact of console time on the perioperative course of RARP. Many authors wrote about possible risk factors that prolong OR time [7,8,9]. However, the effect of operating time on postoperative outcomes including complications, catheter days and length of hospital stay might be understudied. This study involves a monocentric single-surgeon cohort of 500 patients, 40% of which have locally advanced tumors, and aims to examine the effect of longer operating times on functional and oncological outcomes. It also investigates factors which may lengthen an operation.

## 2. Methods

### 2.1. Surgical Procedure and Setting

All procedures (*n* = 500) were completed with Da Vinci X^®^ Surgical Systems (Intuitive Surgical, Sunnyvale, CA, USA). Pelvic lymphadenectomy was performed in all patients due to the risk of upgrading and upstaging, and no intraabdominal drainage was inserted. In the case of urinary tract infection, antibiotics were given according to the urine culture. Otherwise, imperial intravenous single-shot antibiotics with cefuroxime were administered to all patients. Vesicourethral anastomosis (VUA) was carried out in a one-layer fashion with a continuous circumferential. A double-armed barbed suture was used. A Rocco stitch was carried out in the majority of cases in a one- or two-layer fashion. The integrity of the vesicourethral anastomosis was proven via a water tightness test with up to 300 mL of sterile water. Urinary diversion was brought on via both a transurethral (TUC) and a suprapubic catheter (SPC). While the transurethral catheter was removed immediately after a compilation of the anastomosis or on the day after, the suprapubic catheter was removed after an uneventful micturition.

### 2.2. Participants and Methods

A prospectively collected database was established to include 500 consecutive prostate cancer patients who were radically operated on between April 2019 and August 2022 by a specialized robotic surgeon. While 59.4% of tumors were locally confined, 40.6% were locally advanced prostate cancer.

We focused on console time, which was defined as the time from the point when the robot was docked until it was undocked again at the end of the procedure. Regarding the duration of the procedure, patients were divided into three groups based on cut off values of 120 and 180 min: short, under or equal to 120 min; average between 121 and 180 min; long, above 180 min. Those cut-off values were chosen as they were the repeatedly used values in the literature [10,11]. Furthermore, in our clinical practice, we informed our anesthesiology and surgical coordination team about the expected procedural length. We used cutoffs of under 2 h, 2–3 h and above 3 h when a more challenging case due to tumor extension or previous abdominal surgeries was expected. We asked the question of whether or not different durations of the procedure may impact the postoperative course, complications and readmissions. Demographic, intraoperative and postoperative data were analyzed and compared. All postoperative complications within 90 days after the procedure were graded using the Clavien-Dindo Classification [12]. Minor complications were those that were managed conservatively. Major complications were those necessitating an intervention or intensive care or those resulting in organ injury. The length of hospital stay was considered the primary endpoint of the study. Since the transurethral catheter was removed on the day of surgery postoperative day one (POD1), the number of suprapubic catheter days was considered the secondary endpoint. Univariate and multivariate logistic regression models were used to investigate the relationship between console time and various perioperative outcomes. It was also used to identify predictors which could forecast a longer console time.

Statistical analysis was executed using SPSS^®^ v27. Categorical variables were recapitulated as frequencies (percentage) and continuous variables were expressed as mean ± standard deviation, interquartile ranges (IQR) and median values. The Kolmogorov–Smirnov one-sample test was used to confirm normal distribution (test of normality). A one-way ANOVA test was performed for parametric numeric variables. A post hoc comparison (Bonferroni) test was performed in case of a significant ANOVA test result. The independent samples Kruskal–Wallis test was performed for nonparametric variables. Univariable logistic regression and linear regression models were used for further association analysis.

### 2.3. Ethics Statement

The study was directed in accordance with the ethical standards of the Declaration of Helsinki and permitted by the ethics committee of the medical association of Westfalen-Lippe and Wilhelm’s University of Münster (ethical vote: 2022-585-f-S).

## 3. Results

### 3.1. Baseline Parameters

Groups were not statistically different regarding ASA, IPSS and IIEF scores. Tumor parameters such as initial PSA, Gleason score and D’Amico risk classification were also equally distributed among groups. Furthermore, the rate of the nerve-sparing technique was not different between groups (Table 1).

### 3.2. Intraoperative Data

The median docking time was 115, 150 and 215 min in Groups 1, 2 and 3, respectively. The hospital stay was longest in group 3, with a median of 7 days (*p* < 0.001), whereas the median hospital stays were equal in groups 1 and 2 (5 days). Moreover, catheter days were significantly different between groups (*p* < 0.001) with medians of 4, 5 and 7 days in groups 1, 2 and 3, respectively. Stage T3 was highest in group 2 (39.2%), while stage T4 was highest in group 3 (8%). Group 3 patients had the biggest pre-postoperative hemoglobin difference (3 g/dL), groups 1 and 2 patients had comparable pre-postoperative hemoglobin differences (median, 2.5 g/dL). In total, *n* = 368 patients, i.e., 73.6% of patients managed to go home without a suprapubic catheter while the catheter had to be removed in a later outpatient visit after discharge in *n* = 132 patients, i.e., 26.4% of patients. To provide further detail, only 53.4% of men in group 3 compared to 82.2% and 75.3% of men in groups 1 and 2, respectively, left the urological department without catheters. All other intraoperative parameters were comparable among groups. Details can be found in Table 2.

### 3.3. Complications and Readmissions

In total 4.2% of men (*n* = 21/500) required an intervention post-RARP due to adverse events. Patients who underwent longer RARPs experienced more major complications (*p* = 0.008), especially in group 3 (10.2%). Post hoc analysis showed a significant difference only when compared to group 3. Major complications happened similarly between groups 1 and 2 (2.5 vs. 3.1%), while a trend could be suggested for more minor complications in the longer operations group, and statistical analysis showed no difference (*p* = 0.272). Four patients in our cohort suffered from thromboembolic accidents. All of them were in patients operated on longer than 2 h. In total, 5.8% of patients were readmitted within 90 days after RARP with a similar distribution between groups (*p* = 0.338). All anastomosis-related complications such as acute urinary retention [13], urinary tract infection (UTI), vesicourethral anastomosis leakage (VUAL) and upper urinary tract obstruction (UUTO) happened infrequently and were equally distributed among groups. Urinary tract infections may not be directly anastomosis-related, but they were found more often in patients with concurrent vesicourethral anastomosis leakage or micturition disorders, so we included them as anastomosis-related. Upper urinary tract obstructions (UUTO) were found in patients in which the ureter ostium was too close to the anastomosis or an obstruction was observed due to swelling in the anastomosis. Major complications that required interventions could be categorized either as Grade IIIa, and were carried out under local anesthesia, mostly for a symptomatic lymphocele (*n* = 10; 2%). Or as Grade IIIb of whom five men had to be re-operated due to two wound hernias, two cases of ileus and one case of bleeding. Postoperative examination revealed upper urinary tract obstruction in three cases, in which a DJ catheter was inserted under general anesthesia and removed 4–6 weeks later. Details are provided in Table 3.

A univariate regression analysis confirmed our findings that a longer console time increased catheter and hospital days (*p* = 0.012 and <0.001, respectively). However, no correlation was found between console time and readmissions, complications or positive surgical margins. Furthermore console time could not independently predict thromboembolic complications. Details are given in Table 4. Further analysis to predict which parameter could predict longer console times showed a correlation between prostate volume and longer console times (*p* = 0.005). Neither the D’Amico classification, Gleason score, BMI, initial PSA, neoadjuvant hormonal therapy nor pervious surgical treatment of the prostate showed a correlation with console time Table 5.

## 4. Discussion

The core finding of our study is that a longer console time may result in a longer hospital stay and more catheter days. The difference is clearer when the console time exceeds three hours. Console time also correlated to an increased risk of postoperative major complications. Yet, it did not influence minor complications or readmissions. In our study, patients whose surgeries lasted more than three hours stayed one day longer in our urological department (*p* < 0.001) and the urinary diversion through the suprapubic catheter (SPC) was also two days longer (median 7 days) compared to that in patients operated on in less than three hours (*p* < 0.001). Furthermore, we found that patients operated in less than two hours kept their catheter for one day shorter compared to those operated on for more than two hours (median 4 vs. 5 days). On the contrary, Potretzke et al. found that patients who stayed longer in the hospital underwent shorter procedures [14]. In general, the length of hospital stay in our cohort was longer compared to that in studies carried out outside Europe [9,14,15,16,17]. Nevertheless, shorter console time promises a significant reduction in stays and their related costs.

In total, 4.2% of men (*n* = 21/500) in our cohort required an intervention, which is in consistent with the findings in other reports [18]; half of these men belonged to group 3 (*n* = 9/88; 10.2%). While Uchida et al. found no changes in the incidence of serious complications [8], major complications in our cohort were observed more often in men who underwent longer RARPs. This is in line with finding of others [10]. On the other hand, post hoc analysis showed significant differences only when compared to group 3. Major complications occurred similarly frequently between groups 1 and 2 (2.5 vs. 3.1%). Although this might be a coincidental finding, it suggests a threshold of three hours as a surrogate for increased major complications. Nonetheless, and in agreement with Uchida et al. the univariate regression analysis did not show a correlation between console time and major complications [8].

A total of 28 (5.6%) men were readmitted within 90 days after discharge. This is consistent with the findings of other studies [10,19]. Despite the trend of more readmissions in group 3 (8%), statistically there was no difference (*p* = 0.338). While Xia et al. found that longer operating times increased the risk of readmissions, we found no correlation between console time and readmissions (*p* = 0.417). To the best of our knowledge, this is the first study with this number of patients to primarily investigate predictors for prolonged console times and to explore the impact of console time on postoperative outcomes and complications after RARP in a cohort of one surgeon without any exclusion criteria with almost 40% of its patients possessing locally advanced carcinomas.

Due to reimbursement regulations of the national health system, in general, patients in our country tend to stay in hospital preferably until catheter removal after RARP. In our cohort, the overall median catheter and hospital days were 5 days for both. Such a number of catheter days is in the lower range compared to others [5]. Furthermore, we found that only 53.4% of men in group 3 were discharged without a urinary catheter which is significantly lower than the number of men discharged without a urinary catheter in both other groups (82.2% and 75.3% in groups 1 and 2, respectively). Those men had to return for an outpatient visit to have their catheter removed. This suggests a clinically relevant influence of console time on the overall short-term convalescence after RARP. Furthermore, in our study, the rate of overall positive surgical margins (PSM) was relatively low (7.2%). Interestingly, it was equally distributed between groups. On the contrary, Salciccia et al. reported a relation between OR time and positive surgical margins [11]. They found higher PSM rates in men with OR times of less than 120 min compared to those with overall OR times of 172 min. We could not find such a relation between console time and PSM in our univariate linear regression analysis.

In line with the results of others, our univariate analysis validated our findings that a longer console time resulted in a longer hospital stay [11] and more catheter days. Several other studies investigated factors which may lengthen RARP [9,20], however, not all of them found hospital stay to be affected through it [21]. Alenizi et al. found elevated BMI and prostate volume to prolong specific steps of the procedure [7]. In contrast, we found BMI to have no influence on console time [8,20]. On the other hand, increased prostate volume was the only parameter to predict longer console time in our analysis (*p* = 0.005). This is in agreement with the findings of other authors of men with large prostates [20,21]. Wenzel et al. found that a prostate size larger than 40 cc prolonged the OR time but only in a retropubic radical prostatectomy RPE cohort and not in their RARP cohort [20]. Our findings were also confirmed in Retzius-spared robot-assisted radical prostatectomy (rsRARP) cohorts [22].

In our study, *n* = 4/500 (0.8%) men suffered from VTE of which two had a concordant asymptomatic pulmonary embolism. All four incidents happened in groups 2 and 3. This is in agreement with the reports of VTE incidences between 0.5% and 1.8% [1,2].

Nevertheless, according to EAU guidelines [23], not all men must undergo pelvic lymph adenectomy. Yet, in our practice, we perform pelvic lymph adenectomy in all patients to overcome the risk of under-staging or under-grading due to the high rate of tumor misclassification from referring centers.

Other minor complications, especially VUA related ones such as AUR, UTIs or secondary VUAL, did not happen more often in groups 2 or 3. Nevertheless, no conclusions can be drawn due to the small sample size of complications, which presents a limitation of our study.

Complication rate is influenced by tumor characteristics, patient comorbidities, and both the experience and volume of surgeons. We reported complications and readmissions within 90 days after RARP. The amount of total major complications in our cohort was 4.2%, which is consistent with that found by others [24]. Nonetheless, almost 40% of our patients had locally advanced carcinoma, 16.4% had an ASA score of 3 and 6.8% had previous TUR-P which may have complicated the procedure and the postoperative course. Despite preexisting sufficient surgical experience, learning curve effect could not be excluded [25]. However, some complications might be avoided with extensive surgical experience [26,27]. 

The core limitation of our study is its retrospective nature. Furthermore, we did not report the long-term outcomes due to the lack of follow-up data. According to our national health care system, follow up is not conducted by tertiary referral centers or either referring urologists. We reported outcomes, complications and readmissions within 90 days after RARP through a passive follow up, in which we required every referring urologist and general physician to report functional, oncological and other postoperative outcomes of our shared patient.

Furthermore, the learning curve in robot-assisted surgery is not yet exactly defined. In RARP, diverse metrics are used to measure surgical development [25]. Some authors outline the learning curve in RARP according to the threshold of cases and others do so according to the period of time [26]. Yet, improvements in surgical techniques and results are observed even after extensive previous experience [27]. At the beginning of the study, the surgeon possessed almost 500 cases of experience. Nonetheless, this did not exclude a learning curve effect and may bias results as later cases of the procedure in our study might have been completed in shorter time with less complications despite being more challenging.

The duration of postoperative urinary diversion depends on the intraoperative course and the surgeon’s technique, experience and preferences. Additionally, it is affected by patients’ cancer characteristics and medical history. All of mentioned reasons impose a selection bias in our study, which has to be disclosed. In our cohort, men were drained via a suprapubic catheter. Therefore, in the case of micturition disorders or difficulties, it was easier for patients and the treating medical personnel to re-open the catheter and cancel the micturition trial. This may have resulted in longer catheter days in contrast to those of patients drained via a transurethral catheter in the literature [24]. Various factors may prolong the duration of the procedure, such as tumor extent, habitus and previous surgeries as well as the surgeon’s experience [7]. Despite the fact that no significant differences were found in baseline parameters between groups, this did not exclude another selection bias. Clinical surgical results depend on surgeons’ experience and centers’ volume. This study was completed in a tertiary hospital with high sample size and expert surgeon. Therefore the evidence base should be restricted and may not be generalized to other countries or surgeons. Large multicentric, preferably prospective trials, are needed to further investigate our inquiry.

We found that almost half the men (46.7%) operated on for longer than three hours left the hospital with a suprapubic catheter. This may also be due to other reasons, such as advanced tumors and higher rates of serious complications in this group. This is another limitation of our study.

## 5. Conclusions

Longer console times are associated with longer hospital stays, more catheter days and major complications. Patients with shorter operations have better chances at leaving the hospital without catheters. An elevated prostate volume may be a predictor of longer procedures. Yet, even in longer operations, most patients (more than 96%) can expect an uneventful intraoperative and postoperative course. In complicated cases, attention must be paid to avoid postoperative adverse events due to prolonged procedures.

## Figures and Tables

**Table 1:** Analysis of demographic, baseline clinical and preoperative characteristics between groups.

	Total (500)	Short *n* = 157; 31.4%	Middle *n* = 255; 51%	Long *n* = 88; 17.6%	*p*-Value
Age (year)					
Mean ± SD	66.8 ± 7.1	67 ± 6.7	67 ± 6.5	65.7 ± 9	0.260
Median	68	68	68	67	
ASA score					
1	96 (19, 2)	36 (22, 9)	40 (15, 7)	20 (22, 7)	0.777
2	314 (62, 8)	90 (57, 3)	175 (68, 6)	49 (55, 7)	
3	82 (16, 4)	28 (17, 8)	36 (14, 1)	18 (20, 5)	
Missing	8 (1, 6)	3 (1, 9)	4 (1, 6)	1 (1, 1)	
Preoperative HGB (g/dL)					
Mean ± SD	14.7 ± 1.3	14.6 ± 1.1	14.7 ± 1.5	14.9 ± 1	0.613
median	14.8	14.8	14.8	11	
IPSS					
Mean ± SD	11.4 ± 8.3	11 ± 8.3	11.3 ± 8.1	12.9 ± 9	0.383
median	8.3	10	10	11	
IIEF					
Mean ± SD	15.2 ± 8.7	14.6 ± 8.5	15.6 ± 8.8	15.8 ± 7.8	0.261
median	17	17	16	16	
Initial PSA (ng/mL)					
Mean ± SD	14.8 ± 24.5	16.2 ±27.8	13.8 ±20.6	16.5 ± 26.2	0.941
median	8	7.5	8	9.3	
BMI	28.4 ± 4.3 28	27.7 ± 4.5 27	28.8 ± 4.4 28	28.7 ± 4.4 28	0.261
Prostate volume (mL)					
Mean ± SD	49 ± 28	47 ± 23	49 ± 27	53 ± 38	0.236
median	43	44	44	44	
Pre-treatment					
Medical (NHT)	55 (11)	16 (10, 1)	28 (11)	11 (12, 5)	0.881
Surgical (TUR-P)	34 (6, 8)	14 (8, 9)	16 (6, 3)	4 (4, 5)	0.379
D’Amico risk classification					
Low risk	117 (23, 4)	30 (19, 1)	64 (25, 1)	23 (26, 1)	0.627
Intermediate risk	229 (45, 8)	78 (49, 7)	112 (43, 9)	39 (44, 3)	
High risk	154 (30, 8)	49 (31, 2)	79 (31)	26 (29, 5)	
Preoperative Gleason score					
5	1 (0, 2)	1 (0, 6)	0		0.200
6	140 (28)	35 (22, 3)	77 (30, 2)	28 (12, 5)	
3 + 4	176 (35, 2)	63 (40, 1)	80 (31, 4)	33 (37, 5)	
4 + 3	59 (11, 8)	20 (12, 7)	33 (12, 9)	6 (6, 8)	
8	82 (16, 4)	27 (17, 2)	44 (17, 3)	11 (12, 5)	
9	36 (7, 2)	10 (6, 4)	16 (6, 3)	10 (11, 4)	
10	5 (1, 0)	1 (0, 6)	4 (1, 6)	0	
Unclassified	1 (0, 2)	0	1 (0, 4)	0	
Nerve sparing					
Yes	374 (69, 4)	99 (63, 1)	184 (72, 2)	64 (72, 7)	0.548
Partial	19 (3, 8)	12 (7, 6)	3 (1, 2)	4 (4, 5)	
No	134 (26, 8)	46 (29, 3)	68 (26, 7)	20 (22, 7)	

Categorical data are presented as numbers %, SD: standard deviation, ASA: American Association of Anesthesiology comorbidity score, Hgb: hemoglobin, IPSS: International Prostate Symptom Score, IIEF: International Index of Erectile Function, iPSA: initial prostate-specific antigen, BMI: body mass index, NHT: neoadjuvant hormonal therapy, TUR-P: transurethral resection of the prostate.

**Table 2:** Intra- and postoperative data and pathological findings for all groups Group.

	Total (500)	Short *n* = 157; 31.4%	Middle *n* = 255; 51%	Long *n* = 88; 17.6%	*p*-Value
Console time mean ± SD	151 ± 45	109 ± 13	150 ± 17	228 ± 38	
IQR	120–180	100–120	135–160	200–240	<0.001
median	140	115	150	215	
Prostate weight (g)					
Mean ± SD	61 ± 25.6	60 ± 21.9	62.9 ± 25.7	61.2 ± 30	0.546
Median	55	57	57	54	
Pathological stage					
0	1 (0, 2)	0	1 (0, 4)	0	0.027
pT1	1 (0, 2)	0	0	1 (1, 1)	
pT2	295 (59)	91 (58)	147 (57, 7)	57 (64, 7)	
pT3	183 (36, 6)	60 (38, 2)	100 (39, 2)	23 (26, 2)	
pT4	20 (4, 0)	6 (3, 8)	7 (2, 7)	7 (8)	
Postoperative Gleason score					
6	28 (5, 6)	11 (7)	12 (4, 7)	5 (5, 7)	0.217
3 + 4	282 (56, 4)	86 (54, 8)	152 (59, 6)	44 (59)	
4 + 3	89 (17, 8)	23 (14, 6)	46 (18)	20 (22, 7)	
8	26 (5, 2)	11 (7)	10 (3, 9)	5 (5, 7)	
9	29 (5, 8)	10 (6, 4)	11 (4, 3)	8 (9, 1)	
10	1 (0, 2)	1 (0, 6)	0	0	
Unclassified *	45 (9, 0)	15 (9, 6)	24 (9, 4)	6 (6, 8)	
Positive surgical margins	36 (7, 2)	12 (7, 6)	17 (6, 6)	7 (8)	0.892
Number of lymph nodes					
Mean ± SD	19.6 ± 7.4	19.2 ± 7.2	19.7 ± 7.3	20.9 ± 8.1	0.325
Median	18	18	18.5	19	
Positive lymph nodes	87 (17, 4)	26 (16, 6)	48 (18, 8)	13 (14, 8)	0.651
Hgb difference (g/dL)					
Mean ± SD	2.5 ± 4.8	2.5 ± 1.2	2.6 ± 1.38	3.18 ± 1.3	0.001
Median	2.6	2.5	2.5	3	
Transfusion	7 (1, 2)	2 (1, 3)	2 (0, 8)	3 (3, 3)	0.892
Hospitalization (days)					
Mean ± SD	5.6 ± 1.5	5.2 ± 1.1	5.5 ± 1.1	6.4 ± 2.7	<0.001
Median	5	5	5	6	
Catheter days					
Mean ± SD	6.9 ± 4.7	6.2 ± 3.7	6.8 ±4.6	9.1 ± 6.1	<0.001
Median	5	4	5	7	
Suprabubic catheter removed before discharge	368 (73.6%)	129 (82.2%)	192 (75.3%)	47 (53.4%)	<0.001

Categorical data are presented as %; SD: standard deviation, IQR: interquartile range, Hgb: hemoglobin. *: patients received hormonal therapy preoperatively so that a Gleason score labeling postoperatively was not possible by our pathologist.

**Table 3:** 90-day complications and readmissions.

Complications in Detail			Total (*n* = 500)	Short *n* = 157; 31.4	Middle *n* = 255; 51	Long *n* = 88; 17, 6	*p*-Value
	Minor		74 (14, 8)	25 (15, 9)	32 (12, 5)	17 (19, 3)	0.272
Minor	CDI 51 (10, 2)	VTE	4 (0, 8)	0	2 (0, 8)	2 (2, 3)	0.160
		Elevated blood analysis parameters	6 (1, 2)	4 (2, 4)	1 (0, 4)	1 (1, 1)	
		AUR	28 (5, 6)	11 (7)	11(4, 3)	6 (6, 8)	
		Diverse	13 (2, 6)	6 (3, 6)	6 (2, 4)	1 (1, 1)	
	CD II 23 (4, 6)	Secondary VUAL *	11 (2, 2)	2 (1, 3)	7 (2, 7)	2 (2, 3)	
		UTI	11 (2, 2)	2 (1, 3)	4 (1, 6)	5 (5, 7)	
		Hematoma requiring transfusion	1 (0, 2)	0	1 (0, 4)	0	
	Major		21 (4, 2)	4 (2, 5)	8 (3, 1)	9 (10, 2)	0.008
Major	CD III a 12 (2, 4)	Myocardial infarction	1 (0, 2)	0	1 (0, 4)	0	
		Hiatus hernia	1 (0, 2)	0	0	1 (1, 1)	
		Symptomatic lymphocele	10 (2.0)	4 (2, 5)	4 (1, 6)	2 (2, 3)	
	CD III b 8 (1, 6)	Revision	5 (1.0)	0	3 (1, 2)	2 (2, 3)	
		UUTO	3 (0, 6)	0	0	3 (3, 3)	
	CD VI 1 (0, 2)	Rhabdomyolysis	1 (0, 2)	0	0	1 (1, 1)	
		Readmissions	28 (5, 6)	6 (3, 8)	15 (5, 9)	7 (8)	0.338

* Some patients came to emergency with mixed AUR + VUAL + UTIs, and we listed the most serious complaint. Categorical data are presented as %. VTE: venous thromboembolism, AUR: acute urinary retention, VUAL: vesicourethral anastomosis leakage, UTI: urinary tract infection, UUTO: upper urinary tract obstruction. CD III a: complications requiring interventions in local anesthesia. CD III b: complications requiring interventions in general anesthesia.

**Table 4:** Univariate linear regression analysis to determine the relation between operating time and other outcomes.

	Readmission	Minor	Major Complications	Catheter Days	Hospital Stay	Lymphoceles	Positive Surgical Margins	Urinoma	Pulmonary Embolism	Transfusion
console-Time	0.417	0.527	0.569	0.012	<0.001	0.373	0.664	0.234	0.190	0.073

**Table 5:** Univariate linear regression analysis of predictors for prolonged operating time.

	D’Amico Classification	Prostate Volume	BMI	PSA	Gleason Score	Previous Medical Treatment (NHT)	Previous Prostate Surgery
console-Time	0.643	0.005	0.904	0.484	0.274	0.998	0.114

BMI: body mass index, PSA: prostate specific antigen; NHT: neoadjuvant hormonal Therapy.

## Data Availability

Data supporting results is available when requested.

## Abbreviations

ADT	Androgen deprivation therapy
ASA	American association of anesthesiology comorbidity score
AUR	Acute urinary retention
BMI	Body mass index
CD	Clavien–Dindo classification of postoperative complication
HBG	Hemoglobin
IIEF	International index of erectile function
IPSS	International prostate symptom score
ISUP	International Society of Urological Pathology
NHT	neoadjuvant hormonal therapy
NSTEMI	Non-ST-segment elevation myocardial infarction
POD	Post-operative day
PSA	Prostate-specific antigen
PSM	Positive surgical margins
TUR-P	Transurethral resection of the prostate
RARP	Robot-assisted radical prostatectomy
RPE	Retropubic radical prostatectomy
SPC	Suprapubic catheter
TUC	Transurethral catheter
OR-time	Operating time
LOS	Length of hospital stay
UTI	Urinary tract infection
VTE	Venous thromboembolism
UUTO	Upper urinary tract obstruction
VUA	Vesicourethral anastomosis
VUAL	Vesicourethral anastomosis leakage
